# Poles Apart: Where and How Cells Construct Nisin

**DOI:** 10.1128/mBio.02991-20

**Published:** 2020-12-15

**Authors:** Colin Hill

**Affiliations:** a APC Microbiome Ireland and School of Microbiology, University College Cork, Cork, Ireland

**Keywords:** bacteriocins, biosynthesis, nisin

## Abstract

Nisin is a 34-amino-acid lantibiotic that has been used commercially for almost a century as a food preservative. In order to produce active nisin, Lactococcus lactis requires an 11-gene operon that encodes proteins involved in modification, processing, transport, immunity, and regulation. While the role of each of the 11 proteins is well understood, the location and spatial organization of the biosynthetic machinery that involves NisA, NisB, NisC, NisT, and NisP remain to be determined. In this elegant paper (J. Chen, A. J. van Heel, and O. P. Kuipers, mBio 11:e02825-20, 2020, https://doi.org/10.1128/mBio.02825-20), we learn that a NisB dimer is recruited to the “old” pole of a dividing cell, where it assembles with NisC to form a modification complex that can engage with NisA. Unexpectedly, the NisT transporter does not stably assemble into this complex but is distributed around the membrane until it engages with the NisABC complex to transport NisA across the membrane, whereupon it dissociates from NisBC.

## Abstract (precis)

Nisin is a 34-amino-acid lantibiotic that has been used commercially for almost a century as a food preservative. In order to produce active nisin, Lactococcus lactis requires an 11-gene operon that encodes proteins involved in modification, processing, transport, immunity, and regulation.

## COMMENTARY

Nisin is a class 1 lantibiotic and a wonderful example of how the best things sometimes come in small packages. Simply put, nisin is a ribosomally encoded 34-amino-acid antimicrobial peptide with a relatively broad host range. But nisin has many additional features that make it one of the best-studied and most interesting peptides in microbiology. It is used as a commercial ingredient in the food industry to prevent spoilage and foodborne disease (1), it has been used in veterinary medicine to prevent mastitis (2), and it is a signaling peptide that is the basis of the nisin-induced controlled expression system (NICE system) (3). Nisin is enzymatically modified following translation to introduce unusual amino acid residues and five-ring structures, and many natural variants are produced by a range of bacterial species. There is even a range of dedicated nisin resistance mechanisms present in many other species. Nisin is active in the nanomolar range as the result of a mode of action that involves two distinct activities; first, binding to lipid II to prevent cell wall biosynthesis, and second, insertion into bacterial membranes to kill in a manner that does not easily allow resistance to develop (4, 5). The ribosomally encoded nature of nisin and its malleability make bioengineering relatively straightforward, and thousands of variants have been generated and tested for a variety of properties (6). Dutch scientists have led the way in terms of our understanding of nisin, and this paper (7) is yet another wonderful study from the Groningen group led by Oscar Kuipers. This group has consistently contributed some of the most meticulous, painstaking, and insightful science regarding nisin in the last few decades (8).

Two aspects of nisin biology are obviously crucial to its success. First, the peptide must be modified to generate the active lantibiotic in a fashion that presents no risk to the producer cell, and second, the mature nisin peptide must be secreted from the cell. Much is already known about the enzymatic machinery surrounding successful nisin production. The first important step is introducing the modifications that create the lanthionine residues that give the lantibiotics their name. The nisin prepeptide that results from transcription and translation of the structural gene *nisA* must be modified by the dehydration of selected serines and threonines into dehydroalanines and dehydrobutyrines, respectively. This function is performed by a membrane-associated dehydratase, a dimer of NisB, that performs a glutamylation and subsequent elimination to generate the dehydrated residues. Then, the modified peptide undergoes cyclization, a process in which bridges are formed between these newly modified residues and neighboring cysteine residues. This requires the action of a cyclase, a membrane-associated monomer of NisC that directs the correct bridge formation at five sites within the peptide. Next, the prepeptide is exported from the cell by a dedicated ABC transporter, a homodimer of NisT, while the active peptide is finally released from its leader by a protease, NisP.

So, while much is known about the nisin machinery, the cellular localization or spatial organization of its individual components has to date been largely inferred from a series of independent studies to be a membrane-associated multimeric lanthionine synthetase complex composed of NisB (×2), NisC, and NisT (×2). Confirming the existence of such a complex, determining how it is assembled, and identifying its cellular location in live cells were the goals of the study “Subcellular Localization and Assembly Process of the Nisin Biosynthesis Machinery in Lactococcus lactis” by J. Chen, A. J. van Heel, and O. P. Kuipers (7). In a series of elegantly conceived and painstakingly executed experiments involving mutational analysis and fluorescence microscopy, they confirmed that NisB and NisC form a complex localized to the older poles of the cell, while NisT is distributed uniformly around the membrane. The fluorescent images presented in the paper are both informative and beautiful and are a good example of how a picture can be worth a thousand words. While the conclusions can be simply stated, the detailed experimentation is both meticulous and convincing.

The first step was to create a plasmid-based nisin expression system to facilitate the detailed molecular manipulations required to advance the story. Then, they showed that NisA with green fluorescent protein (GFP) fused to the C terminus was correctly modified and displayed good antimicrobial activity once the leader was processed. A series of constructs were also created to generate peptides labeled with FlAsH tags at the N and C termini. The NisA fusions could be shown to bind to NisB and NisC and furthermore confirmed that NisA is localized to the poles of the producer Lactococcus lactis cells. By using a similar fusion strategy, NisB, NisC, and NisT were all labeled with GFP and mCherry at both the N and C termini to generate a series of labeled yet fully functional proteins. These engineered proteins revealed that NisB and NisC are located almost exclusively at the cell poles. A series of time course experiments revealed that NisA, NisB, and NisC all display a singular preference for one of the poles, which was elegantly shown to be the “old” pole. An unexpected finding was that NisT does not appear to assemble into the NisBC complex as had been predicted but is distributed uniformly and circumferentially in the membrane and does not congregate at the poles. This suggests that NisT is recruited to the pole-localized NisBC complex only when and as necessary to complete the secretion of the fully modified prepeptide. To confirm this, a NisT mutant that can no longer secrete nisin was constructed. When this mutation was present, the mutated NisT localized at the pole together with NisB and NisC but could not export the peptide and dissociate. This provides convincing evidence that NisT fleetingly contributes to a NisBCT complex during the secretion process before dissociating and returning to a situation where it is once again distributed around the cell membrane.

The focus then turned to the manner in which the machinery is assembled in the cell. In the absence of NisC and NisT, NisB still localizes to the pole in a fashion similar to that of the native system. However, NisC could localize to the pole only when NisB was present, suggesting that NisB drives the localization at the poles and guides NisC to the correct location. NisA and NisT are not required for this interaction to occur. The fact that NisB and NisC form a complex is entirely consistent with the fact that in class II lantibiotic systems, both dehydration and cyclization are performed by a single protein. Interestingly, in the absence of the NisC cyclase, NisB and NisT can interact at the pole, confirming that NisB is the “glue” that holds the complex together. Finally, a series of truncated NisB derivatives identified a short domain (NisB_750–769_) that appears to be responsible for its targeting to the poles.

**FIG 1:**
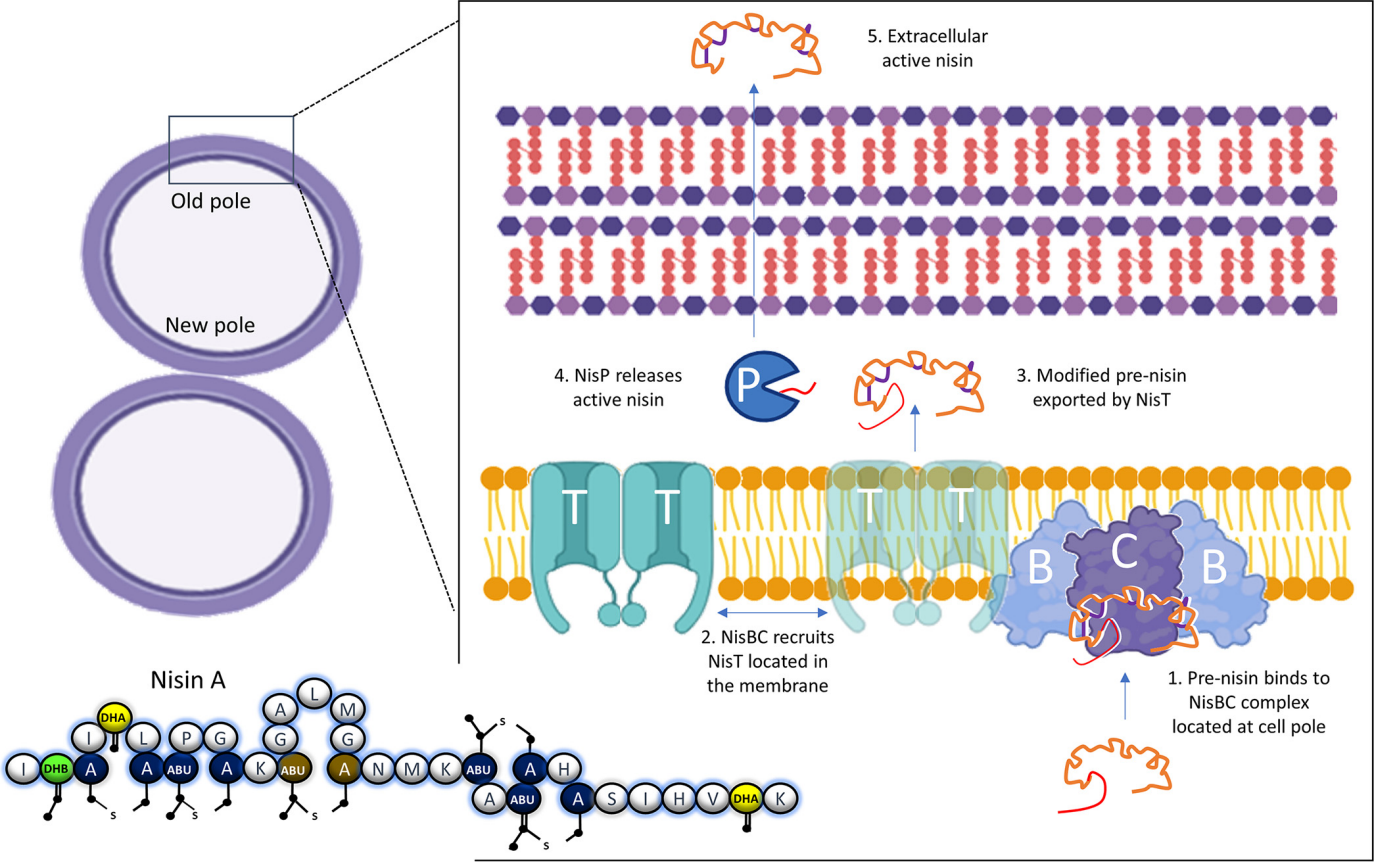
Nisin biosynthesis. NisB, located at the old pole of the cell, recruits NisC to form a NisBC complex. NisA is bound and modified (step 1), and a NisBCT complex is formed by a fleeting interaction with NisT (step 2). The exported, modified nisin (step 3) is then cleaved by NisP before leaving the cell envelope (step 4).

This paper answers many of the open questions surrounding nisin biosynthesis and secretion. It provides a simple and elegant model in which NisB is targeted to the old pole of the producing cell (Fig. 1). The NisB dimer then recruits NisC to form a NisBC complex that can interact with precursor NisA. Once the modifications have been introduced, the complex then recruits two NisT proteins distributed across the membrane in order to secrete the peptide, temporarily forming a NisBCT complex before dissociating again. The authors speculate that this localized production of nisin may be a means of keeping the newly synthesized antimicrobial away from its target lipid II, since there is likely to be less peptidoglycan synthesis and therefore lower concentrations of lipid II at the old pole of a dividing cell. This would reduce the possibility of the cell killing itself during production. One last element remains to be established, in that the precise location or potential role in the NisBCT complex of the extracellular protease NisP was not examined in this study.

The paper advances our fundamental knowledge regarding the very important nisin system, but it also paves the way for future attempts to overproduce nisin and to express other modified peptides. The model developed almost certainly has lessons for researchers studying other secreted peptides.

In an era of multidisciplinary science, where meta-omics is the order of the day and at a time of increasing complexity of scientific studies, this paper is notable for the precision and focus brought to bear on solving this problem by relying on elegantly designed molecular and biochemical methods. It is also noteworthy that only three authors were involved, making this an even more impressive achievement.
